# All-Dielectric Metasurface Lenses for Achromatic Imaging Applications

**DOI:** 10.1186/s11671-022-03720-5

**Published:** 2022-09-02

**Authors:** Menghan Li, Muhan Liu, Yuxuan Chen, Zheng-Da Hu, Jingjing Wu, Jicheng Wang

**Affiliations:** 1 School of Science, Jiangsu Provincial Research Center of Light Industrial Optoelectronic Engineering and Technology, Jiangnan University, Wuxi, 214122 China; 2 State Key Laboratory of Applied Optics, Changchun Institute of Optics, Fine Mechanics and Physics, Chinese Academy of Sciences, Changchun, 130033 China

**Keywords:** All-dielectric metalens, Pancharatnam, Berry phase, Propagation phase, Gerchberg, Saxton algorithm, Holographic phases

## Abstract

Metasurface can use artificial microstructures to manipulate electromagnetic waves more accurately and flexibly. All-dielectric metalens have a wide range of materials and low cost so it has a wide application prospect. Herein, we propose a all-dielectric achromatic metalens built with Si as the structural unit that can operate over a broadband of wavelengths in the visible region. It controls the wavefront of light through the Pancharatnam–Berry phase and propagation phase to eliminate the chromatic aberration. Meanwhile, we also use Gerchberg–Saxton algorithm and its improved algorithm to iterate over multiple design wavelengths and obtain holographic phases suitable for broadband. Thus, both the metalenses and holographic metasurfaces can achieve achromatic broadband in the visible light range, which provides a new method for the development of meta-optical imaging devices.

## Introduction

Metasurfaces are artificial ultra-thin microstructures to easily control electromagnetic waves including their amplitude, phase, and polarization. Compared with traditional optical elements, the advantages of metasurfaces are not only the subwavelength thickness in size, but also the higher accuracy and flexibility from the visible domain [1, 2], infrared domain [3] to the microwave domain [4, 5]. Metasurfaces have excellent potentials in many light modulation applications, such as polarization control [6], metalens [3, 7], vortex-beam generators [8, 9], nonlinear optics [10], color printing [2] and holography [11, 12]. The regulation of electromagnetic waves depended on the electromagnetic resonance effect of the typical metasurfaces, which usually required the noble metals in the structures so that they cause large intrinsic Ohmic loss when applied to the full light wave domain. Hence, all-dielectric metasurfaces [13, 14] are proposed to overcome the loss shortcoming, and they achieve high and uniform transmittance to realize the practical application functions, e.g., the metalens comprises two stacked nanopillar metasurfaces, by which the required focusing phase and dispersion compensation can be controlled independently[15]. More recently, achromatic metalenses [16–19] as the typical subject of metasurfaces could achieve diffraction limited achromatic focusing by modulating optimized amplitude, phase, and polarization for high-resolution holographic imaging [20]. There are many wavefront modulation methods [21] to achieve these significant achromatic metasurfaces, such as transmission phase, geometric phase, resonance phase, or joint control of geometric phase and transmission phase etc. However, there is a great challenge to design an achromatic metalens that is able to eliminate the chromatic effect over a broad band of wavelengths in the visible region to achieve high-performance full-color achromatic imaging applications. Metasurfaces built from traditional optical material Si enable to realize of high-Q resonances in a silicon metasurface with various broken-symmetry blocks [22].

In this paper, we propose an achromatic all-dielectric metalens. We analyze the basic principle of achromatic and the phase modulation method that combines the Pancharatnam–Berry (PB) phase with the propagation phase. We use the phase superposition principle of structural units to construct a metalens that can achromatize in the visible light band, which is composed of a square all-dielectric periodic arrangement of micro-nano units. We compared the focusing results of a metalens using only PB phase modulation with a metalens using both PB phase and propagation phase modulation to verify the effectiveness of using two phase modulations. We also investigate different phase recovery algorithms (PRA) with multiwavelength iterations to achieve achromatic imaging in continuous optical bands. This work will help to broaden the applicable frequency band of holographic phase plates.

## Results and Discussion

**Fig. 1:**
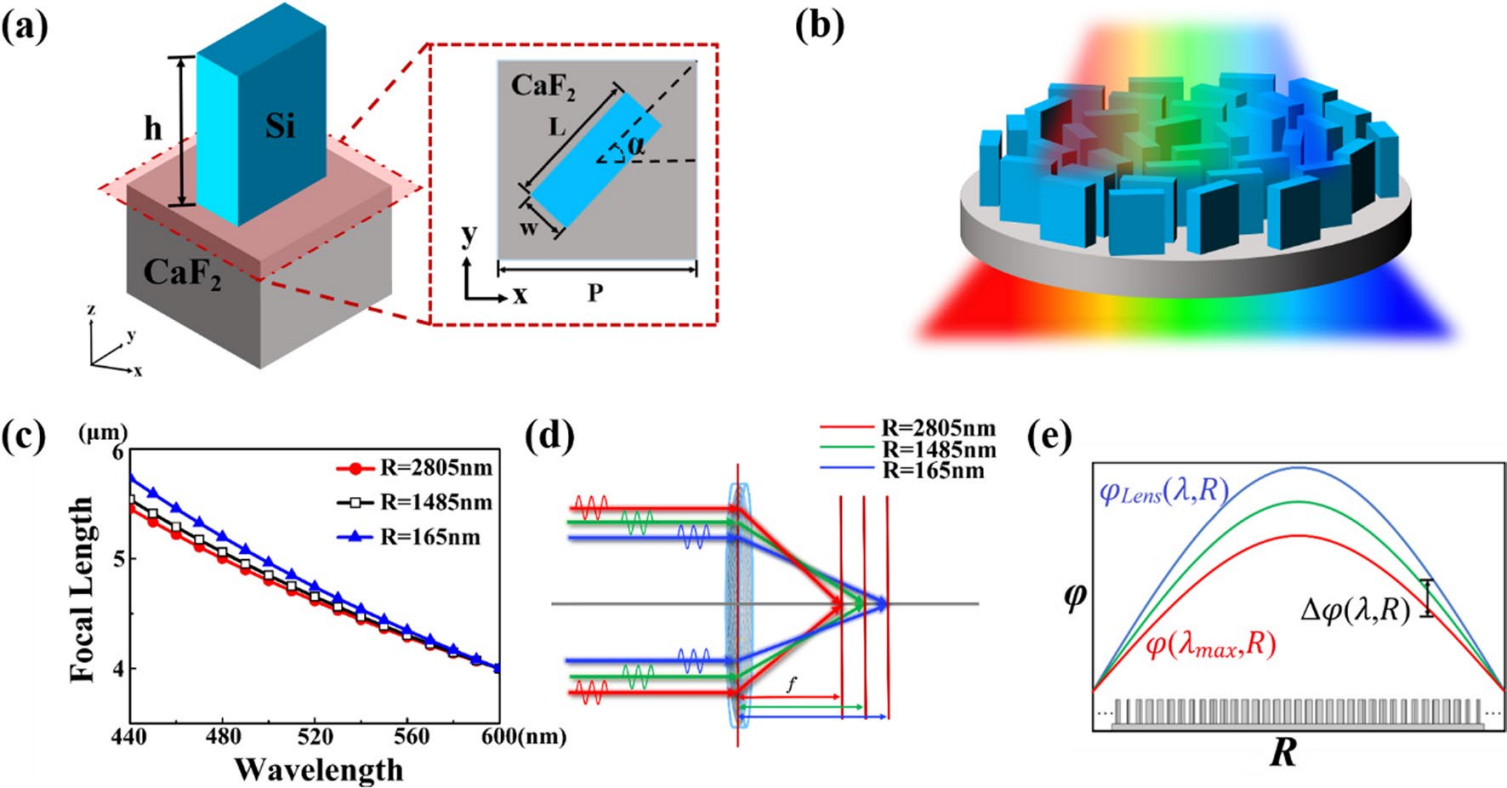
**a** Schematic diagram of the structural unit made of Si and CaF_2_ substrate. **b** Schematic diagram of the metalens. **c** The relationship between the focal length and the wavelength of a lens with a fixed phase distribution. **d** Causes of chromatic aberration: different incident positions of light lead to different focusing positions after passing through the lens. **e** Principle of fixed phase and remaining phase of achromatic metalens

Silicon platforms (SOI) on insulated substrates have the advantages of large refractive index difference, CMOS process compatibility, high density integration, and so on [23]. A schematic of the structural unit of the metalens consists of CaF2 substrate and Si nano-column is shown in Fig. 1a. The height is h, the width is w, the thickness is L, the rotation angle is α, and the period of the structural unit is p. In the visible band, the refractive index of CaF2 is 1.4 and the refractive index of Si decreases with the increase of wavelength. We have taken an approximate value of refractive index in the manuscript. The difference between the refractive index of two materials is instrumental to reduce the reflectivity and ensure a larger adjustable range of effective refractive index of the structure unit in the visible band. We arrange the structural unit array on the circular flat to obtain the achromatic metalens, as shown in Fig. 1b, the whole system is symmetric along the central axis of the circle.

According to Fermat’s principle, light travels along the path of the extreme value. The phase obtained by a plane light passing through a converging lens should satisfy [21]:

1 $$\varphi_{Lens} (\lambda ,R) = - \frac{2\pi }{\lambda }(\sqrt {R^{2} + F^{2} } - F).$$

Among them, λ is the wavelength of the incident light, R is the distance from a point on the lens to the optical axis, and F is the focal length of the lens.

Rewrite the above expression to the explicit expression of F, that is:

2 $$F = \frac{{\varphi_{{\text{Lens }}} (\lambda ,R)}}{4\pi }\lambda - \frac{{\pi R^{2} }}{{\varphi_{{\text{Lens }}} (\lambda ,R)}}\frac{1}{\lambda }.$$

For a definite lens, its phase distribution φ_*Lens*_ is definite and invariable. For a metalens with a theoretical ideal focal point at 600 nm wavelength, as the incident wavelength is gradually blue-shifted, the focal length will gradually increase and the focal spot will expand longitudinally, as shown in Fig. 1c. Besides, the focal length also varies with R in Fig. 1d. These are the causes of chromatic aberration.

The phase delay provided by the metalens can be split into two parts:

3 $$\begin{aligned} \varphi (\lambda ,R) & = \varphi \left( {\lambda_{\max } ,R} \right) + \Delta \varphi (\lambda ,R) \\ & = - 2\pi \left( {\sqrt {R^{2} + F^{2} } - F} \right)\frac{1}{{\lambda_{\max } }} - 2\pi \left( {\sqrt {R^{2} + F^{2} } - F} \right)\left( {\frac{1}{\lambda } - \frac{1}{{\lambda_{\max } }}} \right). \\ \end{aligned}$$

Among them, λ_max_ is the largest wavelength in the incoming composite light. φ is a fixed phase that does not change with the wavelength, and satisfies:

4 $$\varphi \left( {\lambda_{\max } ,R} \right) = - 2\pi \left( {\sqrt {R^{2} + F^{2} } - F} \right)\frac{1}{{\lambda_{\max } }}$$

and Δφ is the remaining phase that is proportional to the reciprocal of the wavelength, it satisfies:

5 $$\Delta \varphi (\lambda ,R) = - 2\pi \left( {\sqrt {R^{2} + F^{2} } - F} \right)\left( {\frac{1}{\lambda } - \frac{1}{{\lambda_{\max } }}} \right)$$

Study proves that metasurface focusing devices can be designed in a PB phase manner, Hasman et.al researchers used the subwavelength grating structure of the rotation angle to modulate the geometric phase [24]. There was a one-to-one correspondence between the rotation angle and the geometric phase value. Based on the Pancharatnam–Berry phase, not only can the amplitude and phase distribution of visible light be fully controlled independently with subwavelength spatial resolution, but also a full complex-amplitude hologram can be realized at visible light wavelengths [25]. Since the PB phase is independent of wavelength, material dispersion, structure size and structure resonance and only related to the rotation angle of the structural unit, so it can be used to achieve the regulation of the fixed phase. Propagation phase is the phase accumulation of electromagnetic waves in structural materials, materials with normal dispersion can be used for adjustment of remaining phase. The physical meaning of the remaining phase is shown in Fig. 1e. Different wavelengths of incident light require different phase distributions to focus on the same focal point. As previously mentioned, the metalens with a fixed phase distribution can’t achieve achromatic.

**Fig. 2:**
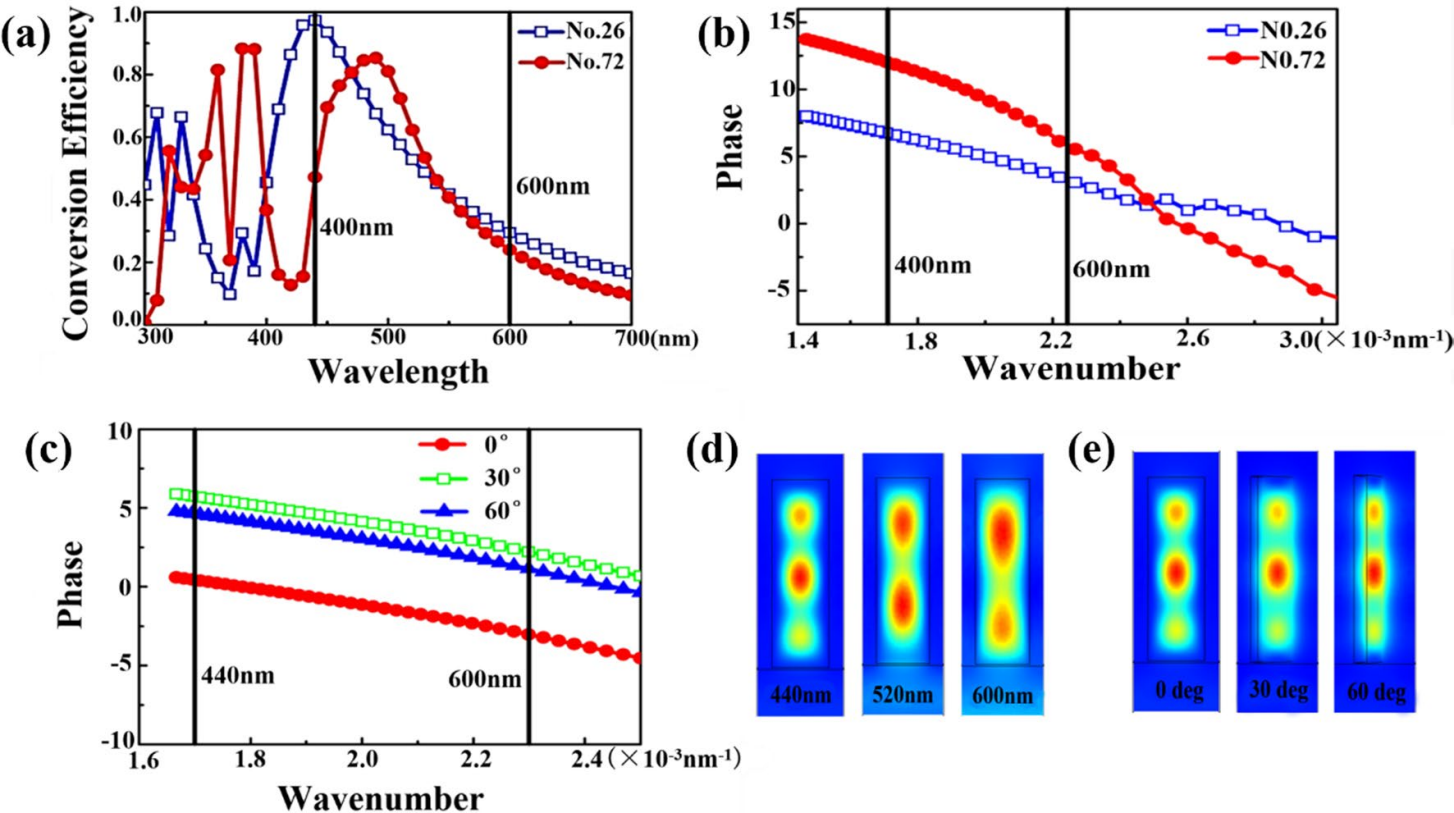
**a** Transmittance spectrum and **b** transmission phase spectrum of structural units under group 26 and 72. **c** Transmission phase spectrum of the corresponding structural units at rotation angles of 0°, 30°, and 60°, respectively. **d** Normalized magnetic field distributions of the corresponding structural units under the 26th group at incident light wavelengths of 440 nm, 520 nm and 600 nm, respectively. **e** Normalized magnetic field distributions of the corresponding structural units at rotation angles of 0°, 30° and 60°, respectively

Herein, we use two phase control methods, PB phase and propagation phase, to realize the superposition of phase delay [26]. Firstly, we obtain the geometrical dimensions (w and L) of the structural unit by calculating the slope required for the propagation phase, and then obtain the rotation angle α by calculating the PB phase. Finally, arrange the array of structural units according to the period p. The value of w is 10, 15, 20,…, 65 nm, the value of L is 90, 95, 100,…,120 nm. Then the parameters are divided into 84 Groups. We choose h = 360 nm and p = 165 nm, and use the comsol software to calculate the transmittance spectrum of the 26th and 72nd group of parameters of the structural unit shown in Fig. 2a.The 26th group parameters were: w = 30 nm, L = 110 nm. Group 72 parameters: w = 65 nm, L = 95 nm. Affected by the material properties, its transmittance in the visible light band decreases rapidly, but remains above 20% at wavelengths of 400–600 nm to ensure the efficiency of metalens.

The dielectric structural unit with heights comparable to wavelength (H–λ) can be regarded as truncated wave-guides [27–30]. In this situation, the phase modulation is based on the propagation phase (also known as the dynamic phase) propagating through the structural unit. The propagation phase is regulated by changing the size of the structural unit, the phase generated by light propagation is:

6 $$\varphi = \frac{2\pi }{\lambda }n_{eff} H$$

where n_eff_ is the equivalent refractive index of the structural unit and H is the height. In principle, phase regulation can be achieved by changing n_eff_ or height H. The phase spectrum of the structural unit is shown in Fig. 2b. In the 440–600 nm band, the transmission phase is linear with the wavenumber (reciprocal of the wavelength) of the incident light, and the phase covers the 2π range, ensuring that the structural unit can achieve achromatic with the compensated phase obtained by the propagation phase method. Figure 2c shows the transmission phase spectrum of the structural units with different rotation angles α can cover the entire 2π interval. When w and L remains unchanged, the slope of the transmission phase spectrum remains basically invariable even if the rotation angle α changes. It proves that the PB phase modulation and the propagation phase modulation are independent of each other. Figure 2d shows the magnetic field of incident waves with wavelengths of 440 nm, 520 nm and 600 nm in the structural unit. Electromagnetic waves of different wavelengths form different resonance modes to ensure high transmittance of the structural unit. Figure 2e shows the magnetic field of the structural unit at different rotation angles, it’s indicated that the rotation angle doesn’t affect the magnetic field distribution.

We use COMSOL to calculate the focusing effect of a one-dimensional microstructure array with 35 structural units arranged at a certain rotation angle. According to Eq. (4), the required phase of the structural unit at different positions of the metasurface is obtained, when F = 4 μm, and the rotation angle of the structural unit is calculated by the following formula:

7 $$\alpha = \frac{{\varphi \left( {\lambda_{\max } ,R} \right)}}{2}.$$

The serial number of the structural unit at the center of the circle is marked as 0, and it is marked as 1 to 17 outwards (with the expansion of the metalens size, the serial number continue to expand). The control parameters of the fixed phase are shown in Table 1.

**Table 1:** Fixed phase control parameters

Serial number	R (nm)	$\varphi \left( {\lambda_{max} ,R} \right)$(rad)	$\alpha$(rad)
0	0	0	0
1	165	− 0.035	− 0.018
2	330	− 0.142	− 0.071
3	495	− 0.320	− 0.160
4	660	− 0.566	− 0.283
5	825	− 0.882	− 0.441
6	990	− 1.264	− 0.632
7	1155	− 1.711	− 0.856
8	1320	− 2.222	− 1.111
9	1485	− 2.794	− 1.397
10	1650	− 3.424	− 1.712
11	1815	− 4.111	− 2.055
12	1980	− 4.851	− 2.426
13	2145	− 5.643	− 2.822
14	2310	− 6.483	− 3.242
15	2475	− 7.370	− 3.685
16	2640	− 8.301	− 4.150
17	2805	− 9.273	− 4.636
…	…	…	…

We calculate the phase spectrum slope that satisfies the structural unit to realize the compensation phase by formula (5), and then find the matching geometric structure parameters(w and L) through the phase spectrum slope. According to Fermat's principle, if the same phase retardation focal length is added to all structural units, the focal length will not change. To facilitate matching of structural units, we can introduce an additive phase *φ*_*shift*_, which is only related to the wavelength and independent of R, and its expression is:

8 $$\varphi_{{\text{shift }}} (\lambda ) = \frac{\chi }{{\frac{1}{{\lambda_{\min } }} - \frac{1}{{\lambda_{\max } }}}}\left( {\frac{1}{\lambda } - \frac{1}{{\lambda_{\max } }}} \right).$$

Among them, χ is the minimum phase difference corresponding to the maximum wavelength and the minimum wavelength of the structural unit.

Then the total phase of the mentalens becomes:

9 $$\varphi_{{\text{Lens }}}^{\prime } (\lambda ,R) = - 2\pi \left( {\sqrt {R^{2} + F^{2} } - F} \right)\frac{1}{\lambda } + \varphi_{{\text{shijt }}} (\lambda ).$$

The added phase is also regulated by the method of the propagation phase, so the remaining phase becomes:

10 $$\begin{aligned} \Delta \varphi^{\prime } (\lambda ,R) & = \Delta \varphi (\lambda ,R) + \varphi_{{\text{shift }}} (\lambda ) \\ & = \left[ { - 2\pi \left( {\sqrt {R^{2} + F^{2} } - F} \right) + \frac{\chi }{{\frac{1}{{\lambda_{\min } }} - \frac{1}{{\lambda_{\max } }}}}} \right]\left( {\frac{1}{\lambda } - \frac{1}{{\lambda_{\max } }}} \right). \\ \end{aligned}$$

The slope of the phase spectrum is:

11 $$k = - 2\pi \left( {\sqrt {R^{2} + F^{2} } - F} \right) + \frac{\chi }{{\frac{1}{{\lambda_{\min } }} - \frac{1}{{\lambda_{\max } }}}}.$$

The significance of this transformation is that the slopes of structural units at different positions can be superimposed on the original theoretical slope on the basis of the minimum slope that the existing structural units can achieve. Assuming that the minimum slope is set as the slope of the structural unit at the center of the circle, the slopes of other structural units only need to be superimposed on this basis. And we don’t need to be limited to a unique value for the theoretical slope, which greatly reduces the slope matching requirements for structural elements.

In addition, since it’s linear for the relationship between the transmission phase of the structural element and the wavenumber, we can also calculate the required slope from the two boundary points at the maximum wavelength and the minimum wavelength:

12 $$k = \frac{{\varphi^{\prime } \left( {\lambda_{\max } ,R} \right) - \varphi^{\prime } \left( {\lambda_{\min } ,R} \right)}}{{\frac{1}{{\lambda_{\min } }} - \frac{1}{{\lambda_{\max } }}}}.$$

In the actual calculation, it should also be noted that since Δ*φ*′ is generally not zero when *λ* = *λ*_*max*_. That is, there is a certain intercept in the regulation of the propagation phase to ensure that the total delay of the propagation phase and the PB phase is equal to $\varphi_{{{\text{Lens}}}}^{\prime }$, then the actual PB phase delay should be equal to:

13 $$\varphi (R) = \varphi_{{{\text{Lens}}}}^{\prime } \left( {\lambda_{\max } ,R} \right) - \Delta \varphi^{\prime } \left( {\lambda_{\max } ,R} \right).$$

Therefore, we can get the geometrical dimensions (w and L) of the structural unit by calculating the slope required for the propagation phase, and then get the rotation angle α of the structural unit by calculating the PB phase that doesn’t vary with wavelength. Finally we can arrange the array of structural units according to the period p.

**Fig. 3:**
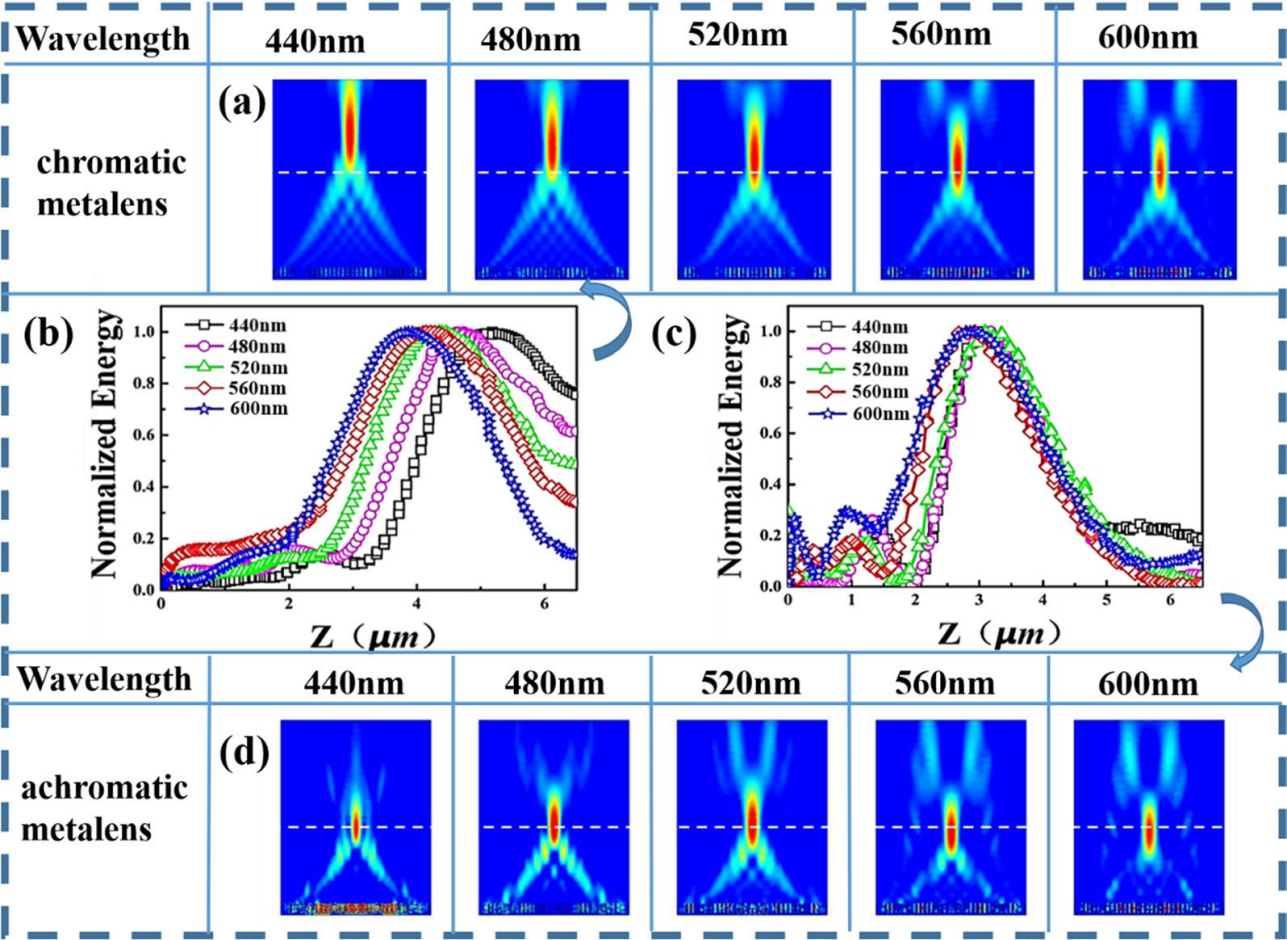
**a** Electric field distribution on the optical axis of a chromatic metalens. **b** and **c** Normalized energy on the optical axis of a chromatic metalens modulated by PB phase only and achromatic metalens modulated by PB phase combined with propagation phase, respectively. **d** Electric field distribution on the optical axis of an achromatic metalens

Here we, respectively, study chromatic metalens modulated by PB phase only and achromatic metalens modulated by PB phase combined with propagation phase. We calculate the focusing effect of a one-dimensional microstructure array with 35 structural units arranged at a certain rotation angle. Figure 3b shows that the electric field intensity distribution curve on the optical axis of the chromatic metalens at different incident wavelengths. It illustrates that F changes with λ and achromatism can’t be achieved. While the electric field intensity distribution curves of the achromatic metalens at different wavelengths shown in Fig. 3c show that the focal length is basically not affected by the incident wavelength. Figure 3a, d shows the motion of the focusing spot under the electric field distribution of the chromatic metalens and achromatic metalens at different incident wavelengths, respectively. It demonstrates that achromatic metalens exhibits higher stability than chromatic metalens. However, since it is difficult for the slope of the achromatic metalens to exactly match the ideal value, it results in some side lobes around the convergence point due to inevitable errors, which affects the image quality to some extent.

Metasurfaces have been widely used to make computer-generated holograms [30–32], and geometric metasurface holograms have achieved diffraction efficiencies of 80% [33]. Further, we use the Gerchberg–Saxton (GS) algorithm to calculate the phase distribution of the holographic plate and explorer its application in achromatic imaging. Gerchberg and Saxton proposed the earliest phase recovery algorithm-the GS algorithm [34]. The GS algorithm calculates the phase delay of holographic plate when the amplitude of image plane is known. The pixels of the calculated phase plate are consistent with the pixels of the input image plane amplitude. However, due to the diffraction of light, the traditional GS algorithm can only obtain the computer-generated hologram for a specific incident wavelength. When the wavelength of incident light is different, aberrations will occur. The weighted Gerchberg–Saxton algorithm (GSW) algorithm is an improvement of GS algorithm, with the same basic concept. The difference is that the GS algorithm uses the expected real image amplitude *B*_0_(*ξ*, *ζ*) to replace the current image real amplitude |*u*_*n*_(*ξ*, *ζ*)|, then get the new image surface wave function:

14 $$\overline{u}_{n} (\xi ,\zeta ) = B_{0} (\xi ,\zeta )\frac{{u_{n} (\xi ,\zeta )}}{{\left| {u_{n} (\xi ,\zeta )} \right|}}.$$

By replacing the current surface real amplitude |*g*_*n*+1_(*x*, *y*)| with the known surface real amplitude *A*_0_(*x*, *y*), a new surface wave function is obtained:

15 $$\overline{g}_{n + 1} (x,y) = A_{0} (x,y)\frac{{g_{n + 1} (x,y)}}{{\left| {g_{n + 1} (x,y)} \right|}}.$$

The GSW algorithm replaces arg[*g*_*n*+1_(*x*, *y*)/|*g*_*n*+1_(*x*, *y*)|] with a linear combination of the phase arg[*g*_*n*-1_(*x*, *y*)/|*g*_*n*-1_(*x*, *y*)|]of the surface at step n-1 and the phase arg[*g*_*n*_(*x*, *y*)/|*g*_*n*_(*x*, *y*) of the current surface at step n to obtain a new surface wave function:

16 $$\overline{g}_{n + 1} (x,y) = A_{0} (x,y)\frac{{g_{n + 1} (x,y)}}{{\left| {g_{n + 1} (x,y)} \right|}}$$

where the parameter γ is a weight factor and 0 ≤ γ ≤ 1. When γ = 1, the algorithm is the GS algorithm [35].

**Fig. 4:**
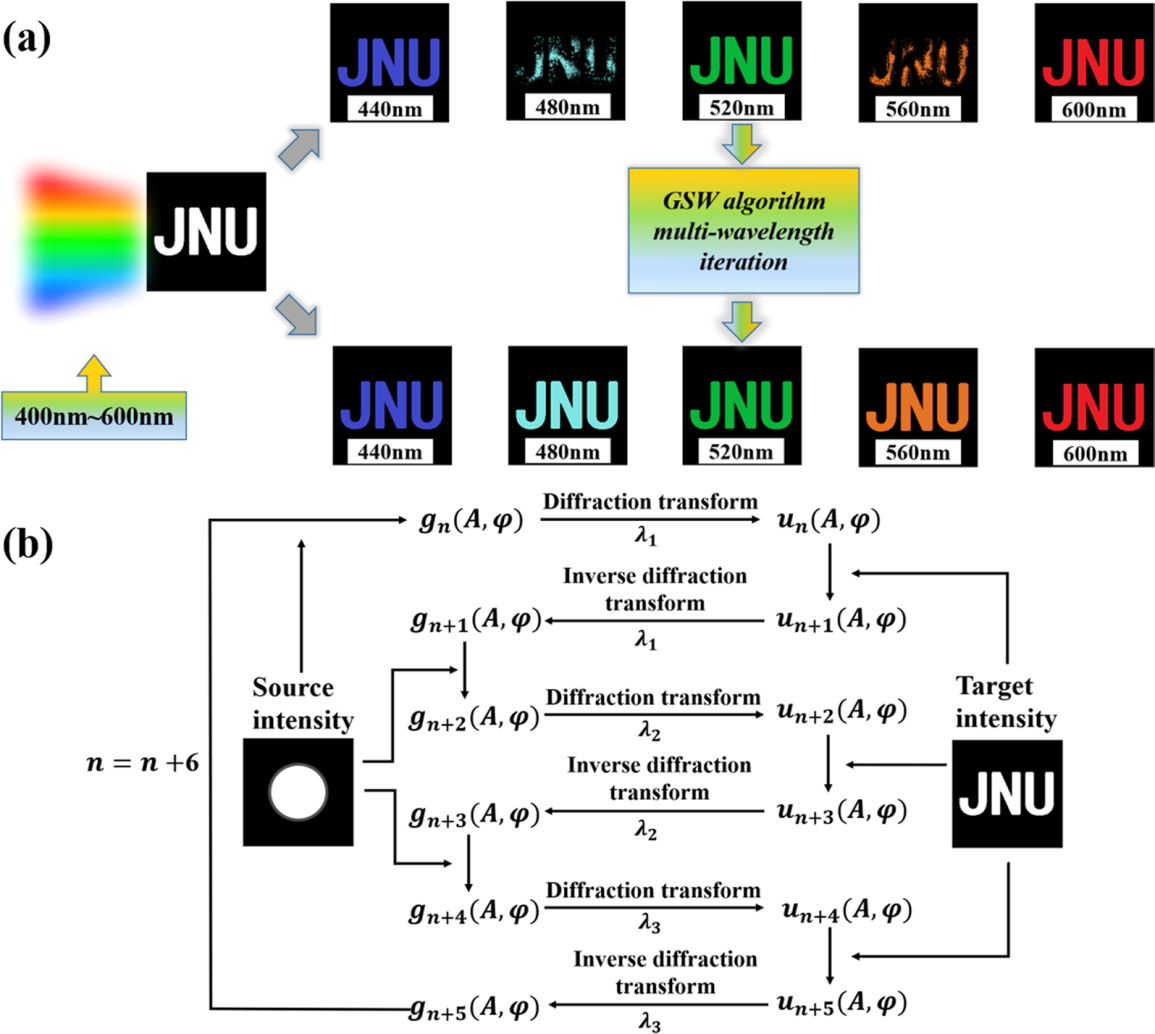
**a** The effect diagrams of “JNU” color hologram imaging are achieved by single-wavelength iteration algorithm (upper row) to multiwavelength iteration algorithm (lower row). **b** Multiwavelength iterative algorithm process for wide-band achromatic

Here let’s take a binary image composed of three letters of “JNU” as an example as shown in Fig. 4a. The methods used as follows: λ_i_ (i = 1, 2, 3) is the three selected design wavelengths. We, respectively, use two PRA at each of these three wavelengths to compute the holographic phase distribution for achromatic imaging.

The theoretical imaging results of the GSW algorithm are shown in the upper row of Fig. 4a. Clear images are obtained at only three design wavelengths while the images between two adjacent design wavelengths is poor. This aberration occurs because the bands between two calculated wavelengths, such as light with wavelengths between 440 and 520 nm, are not considered in the iteration of the algorithm, resulting in chromatic aberration of the image in these bands. The lower image in Fig. 4a was generated using our improved GSW algorithm. In addition to clear images of the three design wavelengths, the bands between design wavelengths are also clearer.

As shown in Fig. 4b, the basic idea of the algorithm is shown, where λ_i_ (i = 1, 2, 3) are the three selected design wavelengths, we perform the PRA on the three wavelengths in turn, then use the obtained phase distribution as the next the initial value of the iterative wavelength. After several iterations, the holographic phase obtained can be applied to the three design wavelengths. The steps of algorithm are as follows:

Step 1: assign the initial value *ϕ*_*init*_ (*x*, *y*) to the object surface phase with known real amplitude, the initial object surface wave function is *A*_0_(*x*, *y*) exp[*iϕ*_*init*_ (*x*, *y*)], the number of iterations n = 0.

Step 2: substitute the object surface wave function *g*_*n*_(*A*, *φ*) into the diffraction integral formula, calculate the wavelength as *λ*_1_, and obtain the image surface wave function *u*_*n*_(*A*, *φ*).

Step 3: replace the real amplitude of the current image surface wave function with the expected image surface real amplitude to obtain a new image surface wave function *u*_*n*+1_(*A*, *φ*).

Step 4: substitute the new image surface wave function into the inverse diffraction integral formula, calculate the wavelength as *λ*_1_, and obtain the object surface wave function *g*_*n*+1_(*A*, *φ*).

Step 5: replace the real amplitude of the current object surface wave function with the known real amplitude of the object surface to obtain a new object surface wave function *g*_*n*+2_(*A*, *φ*).

Step 6: set the calculation wavelength to *λ*_2_, make n = n + 3, and repeat steps 2 to 5.

Step 7: set the calculated wavelength to *λ*_3_, make n = n + 3, and repeat steps 2 to 5.

Step 8: go back to the second step, start a new iteration, until n/6 equals the preset total number of iterations, the automatic loop terminates. We can calculate the light intensity distribution in the observation plane through *I*_*n*_ =|*u*_*n*_(*A*, *φ*)|^2^ and obtain the phase of the holographic plate through *ϕ*_*n*_(*x*, *y*) − *ϕ*_0_(*x*, *y*).

**Fig. 5:**
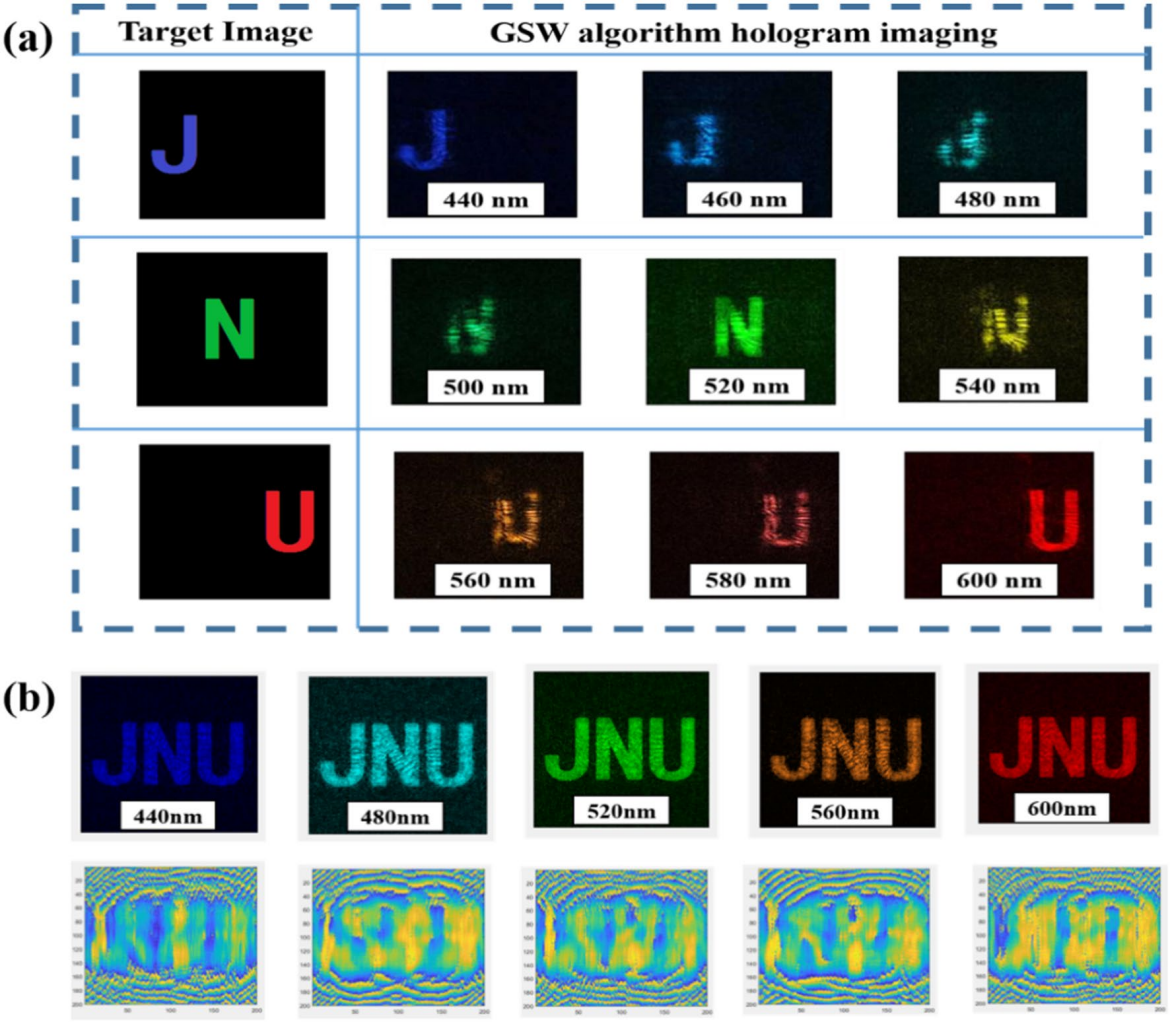
**a** The resulting image of the GSW algorithm for a single wavelength iteration when incident on the three letters of “JNU” at 440 nm, 520 nm, and 600 nm, respectively. **b** Multiwavelength GSW iterative realization of “JNU” color image effect and phase map

Next, we select three design wavelengths, perform PRA on the three wavelengths in turn, and use the obtained phase distribution as the initial value of the next iteration wavelength. After many iterations, the obtained holographic phase can be applied to this three design wavelengths. In order to achieve color imaging in the 440–600 nm band, let λ_1_ = 440 nm, λ_2_ = 520 nm, λ_3_ = 600 nm, and the real amplitude distributions of the image plane under a single wavelength are letters: “J,” “N” and “U,” the calculation result is shown in Fig. 5a. Obviously, the image is relatively clear only at three design wavelengths, but imaging is poor for incident wavelengths between the two design wavelengths. When the incident wavelengths are 480 nm and 560 nm, the image is severely distorted, letters cannot be recognized, and the image position is in a transitional stage. When λ_1_ = 440 nm, λ_2_ = 520 nm, and λ_3_ = 600 nm, the real amplitude distribution of the image plane at a single wavelength is the combination of the letters "JNU," and the calculation result of the GSW algorithm is shown in Fig. 5b. We can see that the images at the three design wavelengths are clear, and the images of the bands between the design wavelengths are also clearer and easier to identify. There is no noticeable noise in the background, which hardly affects the final image quality of panchromatic imaging. The results are in perfect agreement with the theory in Fig. 4.

## Conclusions

In conclusion, we design a visible band all-dielectric metalens and analyze the structure unit of the metalens. Firstly, we study the effects of its geometric parameters and rotation angle on the transmission and phase of the incident light. According to the principle of phase overlay, the PB phase is combined with the propagating phase to achieve the fixed phase control and the remaining phase control, respectively. The final calculation shows that the focal length of the achromatic metalens does not change with the wavelength of the incident light in the range of 440–600 nm and achromatic effect is pretty excellent. Then we introduce the improved GSW algorithm with multiple wavelengths to achieve achromatic imaging in the visible band. Using GSW method for achromatic imaging of polychromatic light, we find that it has better imaging quality in the whole band. Therefore, the achromatic holographic metasurfaces generated by GSW method can widen the applicable frequency band of the holographic phase plate.

## Data Availability

The datasets and figures used and analyzed during the current study are available from the corresponding author on reasonable request.

## Abbreviations

PB: Pancharatnam–Berry phase
PRA: Phase recovery algorithms
GS: Gerchberg–Saxton algorithm
GSW: The weighted Gerchberg–Saxton algorithm
